# Supplementary material for: HIVprotI: an integrated web based platform for prediction and design of HIV proteins inhibitors
Source: J Cheminform. 2018 Mar 9;10:12. doi: 10.1186/s13321-018-0266-y (PMC5845081; doi:10.1186/s13321-018-0266-y)
Supplement: Supplementary file 2 — Additional file 2. Source code of HIVProtI web server. [file 13321_2018_266_MOESM2_ESM.zip › HIVprotI_Source-code/hivprotI/weka-3-6-6/doc/weka/attributeSelection/AttributeSetEvaluator.html]

AttributeSetEvaluator


---


|  |  |  |
| --- | --- | --- |
| **Overview** \| **Package** \| **Class** \| **Tree** \| **Deprecated** \| **Index** \| **Help** \| **Weka's home** | |  |
| **PREV CLASS**   **NEXT CLASS** | **FRAMES**    **NO FRAMES**     **All Classes** |  |
| SUMMARY: NESTED \| FIELD \| CONSTR \| METHOD | DETAIL: FIELD \| CONSTR \| METHOD |  |


---


## weka.attributeSelection Class AttributeSetEvaluator

```
java.lang.Object
  weka.attributeSelection.ASEvaluation
      weka.attributeSelection.AttributeSetEvaluator
```

**All Implemented Interfaces:**: java.io.Serializable, CapabilitiesHandler, RevisionHandler

---

``` public abstract class AttributeSetEvaluator extends ASEvaluation ```

Abstract attribute set evaluator.

**Version:**
:   $Revision: 1.3 $

**Author:**
:   Zheng Zhao: zhaozheng at asu.edu

**See Also:**: Serialized Form

---

**Constructor Summary**

`AttributeSetEvaluator()`


| **Method Summary** | |
| --- | --- |
| `abstract  double` | `evaluateAttribute(int attribute)`             evaluates an individual attribute |
| `abstract  double` | `evaluateAttribute(int[] attributes, int[] classAttributes)`             Evaluates a set of attributes |

**Methods inherited from class weka.attributeSelection.ASEvaluation**

`buildEvaluator, forName, getCapabilities, getRevision, makeCopies, postProcess`

**Methods inherited from class java.lang.Object**

`equals, getClass, hashCode, notify, notifyAll, toString, wait, wait, wait`

**Constructor Detail**

### AttributeSetEvaluator

```
public AttributeSetEvaluator()
```


**Method Detail**

### evaluateAttribute

```
public abstract double evaluateAttribute(int attribute)
                                  throws java.lang.Exception
```

:   evaluates an individual attribute

    :   **Parameters:**: `attribute` - the index of the attribute to be evaluated **Returns:**: the "merit" of the attribute **Throws:**: `java.lang.Exception` - if the attribute could not be evaluated

---


### evaluateAttribute

```
public abstract double evaluateAttribute(int[] attributes,
                                         int[] classAttributes)
                                  throws java.lang.Exception
```

:   Evaluates a set of attributes

    :   **Parameters:**: `attributes` - an `int[]` value: `classAttributes` - an `int[]` value **Returns:**: a `double` value **Throws:**: `java.lang.Exception` - if an error occurs


---


|  |  |  |
| --- | --- | --- |
| **Overview** \| **Package** \| **Class** \| **Tree** \| **Deprecated** \| **Index** \| **Help** \| **Weka's home** | |  |
| **PREV CLASS**   **NEXT CLASS** | **FRAMES**    **NO FRAMES**     **All Classes** |  |
| SUMMARY: NESTED \| FIELD \| CONSTR \| METHOD | DETAIL: FIELD \| CONSTR \| METHOD |  |


---
